# Comparative outcomes of foot cast and short leg cast in pseudo-Jones avulsion fracture: a single blinded randomized controlled trial

**DOI:** 10.1186/s13047-019-0359-5

**Published:** 2019-09-10

**Authors:** Peerapong Piyapittayanun, Kanakij Mutthakalin, Alisara Arirachakaran, Jatupon Kongtharvonskul

**Affiliations:** 1 Orthopedics Department, Police General Hospital, Bangkok, Thailand; 2 Orthopedics Department, Bumrungrad Hospital, Bangkok, Thailand; 3 Sport and orthopedic center, Samitivej hospital and Section for Clinical Epidemiology and Biostatistics, Faculty of Medicine Ramathibodi Hospital, Bangkok, Thailand

## Abstract

**Background:**

Fractures of the metatarsal bones account for 35% of all foot fractures. Conservative management of fractures proximal to the metaphyseal-diaphyseal junction of the fifth metatarsal bone (pseudo-Jones) is by protected weight bearing. The methods of protected weight bearing include short-leg casting and splinting (boot cast, Jones’s bandage and elastic bandage). However, currently there is no consensus as to which method is the most suitable.

**Method:**

We have conducted a randomized controlled trial to compare outcomes of foot casting (FC) and short leg casting (SLC) to assess pain, function and complication outcomes for the treatment of pseudo-jones metatarsal fractures. This single-center, single blind,randomized controlled trial was conducted between 1 June 2016–1 July 2018 at Police General Hospital, Bangkok, Thailand.

**Result:**

A total of 72 pseudo-jones metatarsal fracture participants were randomly allocated to treatment by foot cast or short leg cast. The primary outcomes were pain VAS, AOFAS and complications measured at 2, 4, 6 and 8 weeks after receiving the treatment. Seventy-two patients (36 paticipants per group) were enrolled to receive either FC or SLC. The mean VAS measured at baseline, 2 weeks, 4 weeks, 6 weeks and 8 weeks were 7.36, 1.97, 0.58, 0.17 and 0.08 respectively in the FC group; and 6.09, 2.91, 1.23, 0.37 and 0.11 respectively in the SLC group. The mean AOFAS at baseline, 2, 4, 6 and 8 weeks were 33.60, 68.22, 82.72, 91.75 and 98.11 respectively in the FC group; and 32.60, 60.20, 70.20, 92.24 and 99.13 in the SLC group. The estimated mean difference of pain VAS and AOFAS at 2 weeks and 4 weeks were − 0.94 (95% CI: − 1.53, − 0.34), − 0.65 (95%CI: − 1.24, − 0.05), 8.02 (95%CI: 3.74, 12.10) and 12.52 (95%CI: 8.27, 16.78), which were statistically significantly better in the FC groups when compared to the SLC groups. However, there were no statistically significant difference between the two groups at 6 and 8 weeks.

**Conclusion:**

This study demonstrated that the application of foot casting can improve pain VAS and AOFAS function at 2 and 4 weeks in the treatment of pseudo-jones metatarsal fractures when compared to short leg casting. However, at 6 and 8 weeks, there were no statistically significantly different between the two groups.

## Introduction

Fractures of the base of the fifth metatarsal are a common injury originally described by Sir Robert Jones in 1902 [1, 2]. Since then, virtually all fractures involving the proximal aspect of the fifth metatarsal have been classified as “Jones” fractures. Several authors, however, have recognized the existence of at least two major patterns of fracture at the base of the fifth metatarsal: (1) an avulsion fracture of a variably sized portion of the tuberosity or the most proximal part of the metatarsal; and (2) a transverse fracture through the proximal diaphysis of the metatarsal within 1.5 cm of the tuberosity, which has been called a “pseudo-Jones avulsion fracture” [1–11]. Several methods of non-operative treatments have been studied, including elasticated bandaging and wearing a hard-soled shoe, through to immobilization in a cast, focused rigidity casting or a walking boot [2, 4, 12–19].

**Fig. 1:**
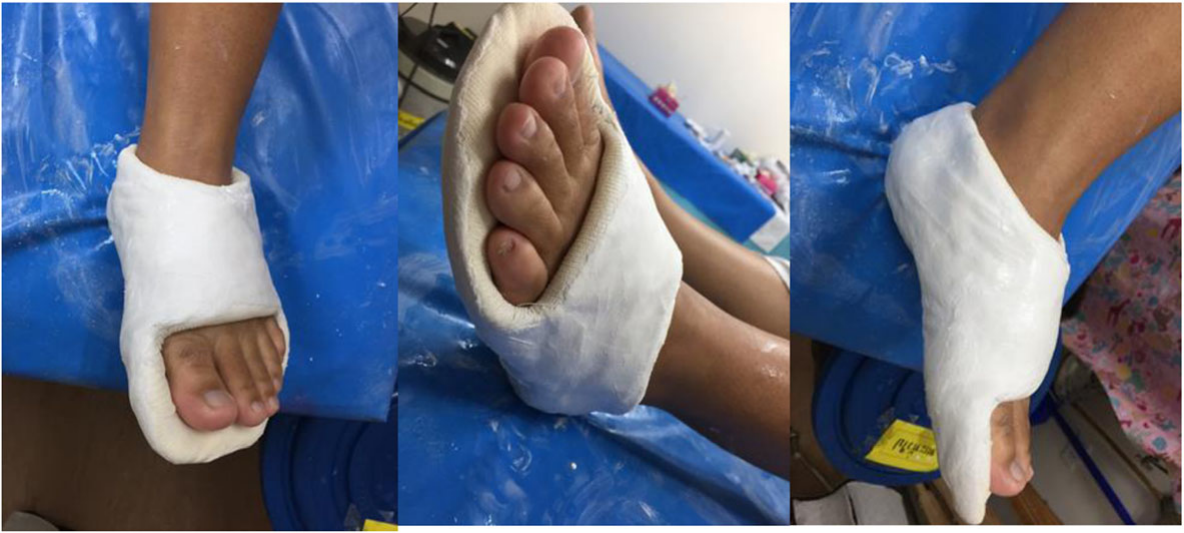
This figure showed the foot cast that treatment fracture by casting is immobilize one joint above and one joint below which is tarsometatarsal joint and metatarsophalangeal joint to treat metatarsal fracture

Several comparative studies have compared short leg casting and splinting (elasticated or compression bandaging and walking boot) [2, 14, 17–19]. However, no consistent results have been provided in these published trials. Only one previous meta-analysis [20] reported that for foot function outcomes (1 month or more), foot splinting had higher function than short leg casting and lower non-union rates in the treatment of acute avulsion fracture fifth metatarsal bone. However, foot splinting had higher pain scores at 1 month when compared to short leg casting. The explanation of this result is because short leg casting had better rigid stabilization, resulting in lower nonunion rates and pain scores for fractures of the fifth metatarsal bone when compared to foot splinting. However, the rigid stabilization of short leg casting results in limited ankle motion and lower foot functional scores. The concept of treatment of the fracture by casting is immobilization one joint above and one joint below, which is the tarsometatarsal joint and the metatarsophalangeal joint to treat metatarsal fracture, therefore the foot cast (Fig. 1) that covers both joints with the ankle joint having full motion would be better to treat it. Therefore, the foot cast should be applied to treat fractures of the fifth metatarsal bone and will improve pain, function and prevent nonunion fracture. The aims of the present study were to compare short leg casting and foot casting for treatment of pseudo-Jones avulsion fractures proximal to the metaphyseal-diaphyseal junction of the fifth metatarsal bone.

## Material and method

### Trial design

The study design was a single-blind randomized controlled trial, which was conducted at the Orthopedics outpatient clinic, Police General Hospital, Bangkok, Thailand during June 2016 – July 2018. Informed consent was obtained from all study participants. Approval for the study was given by the Committee on Human Rights Related to Research Involving Human subjects at the Police General Hospital, and the protocol (ID 53/2560) of the study was registered at ClinicalTrials.gov (NCT03170687).

### Participants

Adult patients who presented within seven days of injury with a closed pseudo-Jone fracture were considered for inclusion. The degree of displacement or comminution, or the propagation of fracture into the fifth tarsometatarsal joint did not preclude recruitment into the trial. All participants were willing to participate and provided consent. Exclusion criteria included open fracture; multiple fractures; nonunion; delayed union; pathological fracture; bone tumor; diabetes; inflammatory joint disease; previous ipsilateral foot surgery or fracture; presentation more than seven days after the injury and an inability to understand written English. An information sheet about the study was given to participants in the Emergency Department or in the out-patient department, which allowed at least 24 h, during which the patients could agree to participate. Recruitment into the study with a further explanation and informed consent was carried out in the clinic.

### Treatment regimen and randomization

**Fig. 2:**
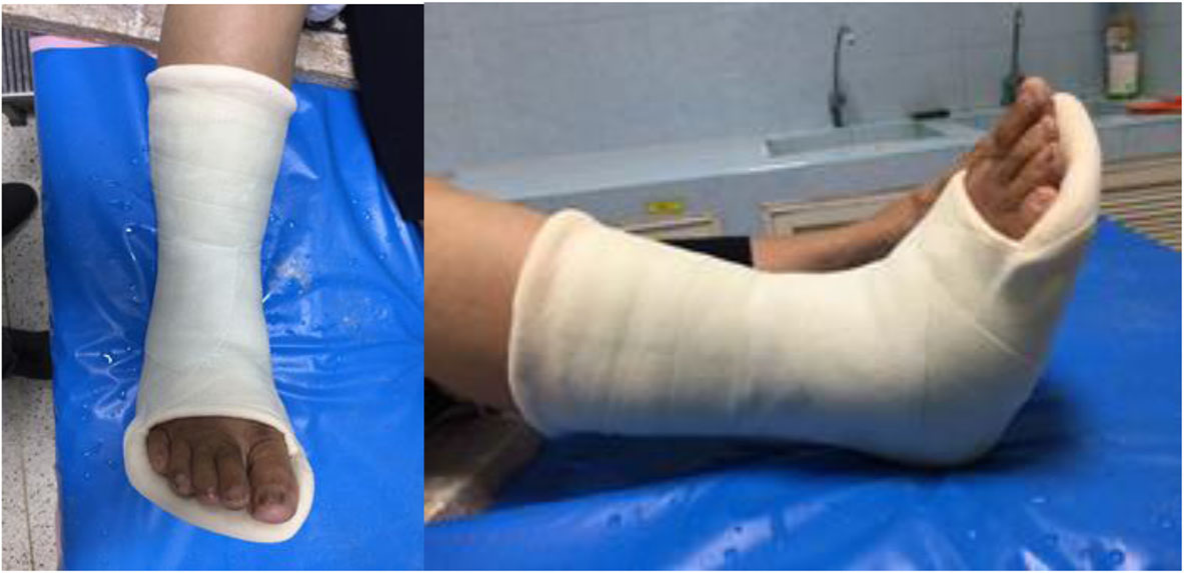
This figure showed the plaster of Paris below knee cast

Eligible participants were randomly assigned to wear a foot cast (Fig. 1) applied by well-trained orthopedic staff or resident. The foot cast consisted of alternative layers of cast padding and plaster of Paris cast with a total of four or five layers of each applied. The determined landmark of casting of the foot cast started from about 1 cm proximal to the base of the metatarsal bone (anterior), under the tip of medial malleolus (medial), the lateral malleolus (lateral) and 2 cm above the Achilles tendon insertion (posterior). The end of the foot cast is distal to the head of the metatarsals and all toes should be clearly visible (Fig. 1). The other group was given a plaster of Paris below knee cast (Fig. 2) applied by well-trained orthopedic resident or staff. All participants in both groups of treatment were for four weeks. The use of elbow crutches was permitted in both groups and patients were encouraged to bear weight as soon as they could tolerate it. All participants had cast removed four weeks later in the clinic. Participants in this group were evaluated for the risk of venous thromboembolism and offered prophylaxis according to our Trust guidelines.

A block randomization with a ratio of 1:1 was applied to generate a randomization list, with varying block size of 4. This was done by the biostatistician (J.K.), who was not involved in the participants’ recruitment or data collection. STATA version 14.0 software [21] was used to generate the random sequence lists [21]. Envelopes were opened in numerical order immediately before administering intervention. Assessors and researchers did not know which one was FC or SLC. Participants might be prescribed other pain relief (acetaminophen 500 mg or NSAIDs) depending on the physician’s judgement. The use of NSAIDs could be started with ibuprofen (400 mg) 1 tablet three times per day or naproxen (250 mg) 1 tablet two times per day if participants were allergic to ibuprofen. The patients were provided with a diary to record their daily pain medication intake.

### Outcome measures

The primary measurement tools were the American Orthopedic Foot and Ankle Score (AOFAS) including four rating systems were developed by the American Orthopaedic Foot and Ankle Society in 1994 to provide a standard method of reporting clinical status of the ankle and foot. The systems incorporate both subjective and objective factors into numerical scales to describe function, alignment, and pain [22]. The AOFAS is a disease-specific quality of life questionnaire designed for participants with disorders of the four different regions of the foot: the ankle-hindfoot, midfoot, metatarsophalangeal (MTP)-interphalangeal (IP) for the hallux, and MTP-IP for the lesser toes [22]. The AOFAS (ranging from 0 to 100 which the higher score indicated greater function) consists of nine questions and covers three categories: pain (40 points), function (50 points) and alignment (10 points). These are all scored together for a total of 100 points. This study use AOFAS validated in Thai version which have the same construct with original AOFAS [23]. Treatment efficacy was evaluated by a trained research assistant at pre-casting, post-casting, 2 weeks, 4 weeks, 6 weeks and 8 weeks after casting. Secondary outcome measures were pain score measured using a VAS (ranging from 0 to 10 which the higher score indicated greater pain). A trained research assistant measured the VAS score at baseline, 2 weeks, 4 weeks, 6 weeks and 8 weeks after treatment. In addition, adverse events including soft tissue irritation (presence of callosity, problems finding appropriate footwear and sensory disturbance in the foot), delayed union, nonunion, and re-fracture were also assessed at each visit after casting. Other co-variables including age, gender, underlying and disease severity at baseline were also collected.

### Statistical analysis

Data were described using frequency for categorical data, and mean (SD) or median (range) where appropriate for continuous data. The baseline characteristics were then explored. The baseline characteristics were then explored. If their distributions were different between the two intervention groups, i.e., ≥ 10% for binary/categorical variable and ≥ 1 of the pooled SD for continuous variables, these variables were then considered for adjusting in the main analysis.

We compared the continuous outcomes; AOFAS and pain VAS between intervention groups using a two-sample t-test. Secondary analysis was a mixed linear regression analysis with hierarchical approach, in which a subject-variation term was fitted in the model as a random effect and treatment groups was considered as a fixed effect. In addition, times at measurement were also included in the mixed model by adding interaction effect of treatment and time (i.e, treatment x time) for repeated measurements per participant. Marginal treatment effects between treatments and times were then estimated and compared. Co-variables at baseline were included if they were unequally distributed between two groups. The normality of residuals of the mixed model was then checked using normality plots (i.e., quantile of normal distribution) and the Shapiro-Wilk test. Diagnostic measures were explored if the assumption of normality was violated. The continuous outcomes were then transformed where appropriate to meet the assumption.

An intention-to-treat analysis (ITT) approach was applied for all analyses if there was any evidence of a protocol violation. All analyses were performed using STATA version 14.0 [21]. Bonferroni correction was applied to adjust for inflation of type one error from six outcomes and thus 4 multiple tests [24]. If a significance level for the whole family of tests was 0.05, then the Bonferroni corrected threshold for individual test was 0.0125.

### Power calculation

The sample size was calculated to detect a mean difference in AOFAS between foot cast and short leg cast. For the meta-analysis [20], the mean and standard deviation (SD) of AOFAS scores in the short leg cast group were 87.9 and 11.55 respectively. Type I error, power of test, and ratio of the treatment groups were set at 0.05, 0.80, and 1:1 respectively. The estimated sample size was 28 for each group to detect the mean difference of AOFAS of 9 units [25]. Loss to follow up was estimated at 20%, which yields a required sample size of 36 participants.

## Results

### Patient characteristics

**Fig. 3:**
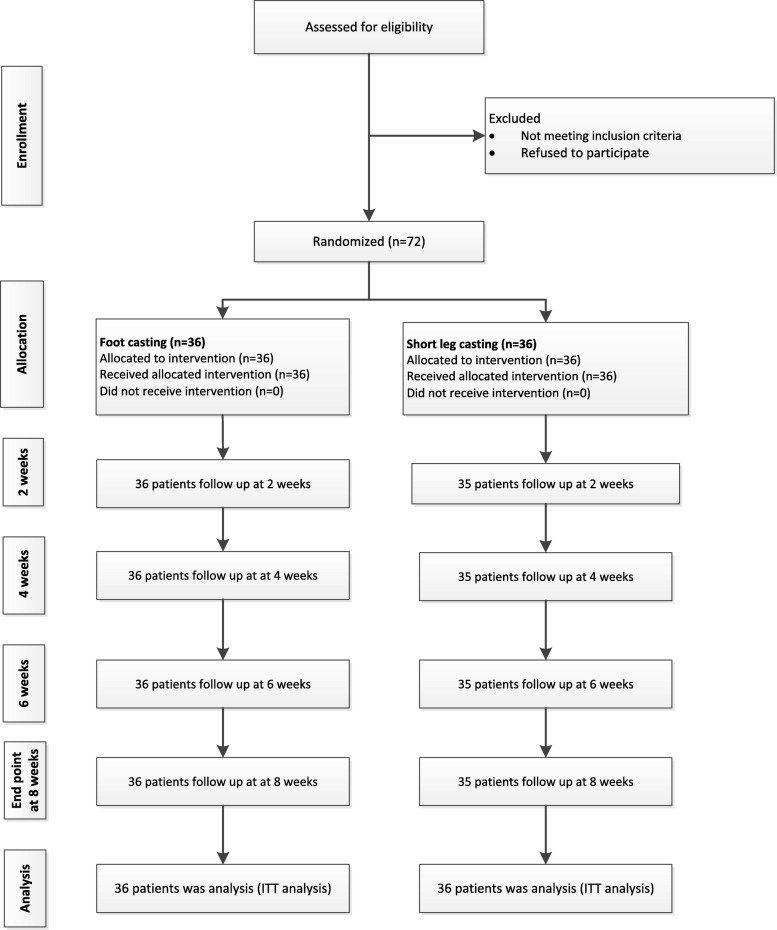
Consort 2010 Flow diagram

A total of 72 participants were recruited and randomly allocated to treatment groups, see Fig. 3 and Table 1. Baseline characteristics were described and compared between treatment groups, see Table 1. For the FC group, the majority were female 26 (72.2%), with a mean (±SD) age of 41 (±16.1) and mean time after injury of 2.03 (1–6) hours. The corresponding characteristics in SLC group were female 12 (±33.3%), 40.1 (±13.5) years and mean time after injury of 6.67 (1–24) hours. Participant’s compliance with the allocated treatments was 100% in both groups, measured by assessed cast at each visit. Compliance was 100 and 97% in FC and SLC group, respectively.

**Table 1:** Baseline characteristics of patients between treatment groups

Characteristics	Foot cast (*n* = 36)	Short leg cast (*n* = 36)
Age (year), mean (SD)	41 (16.1)	40.1 (13.5)
Sex (%)		
Male	10 (27.8)	24 (66.7)
Female	26 (72.2)	12 (33.3)
Time after injury (hour), mean (range)	2.03 (1–6)	6.67 (1–24)
Pain VAS score, mean (SD) (0–10)	7.36 (1.57)	6.09 (1.77)
AOFAS, mean (SD) (0–100)	33.6 (12.49)	32.6 (12.64)
- Pain, mean (range) (0–40)	6.11 (0–20)	6.29 (0–20)
- Function, mean (range) (0–45)	5.11 (0–16)	5.08 (0–14)
Activity limitations, mean (range) (0–10)	2.08 (0–5)	2.29 (0–5)
Maximum walking distance, mean (range) (0–10)	0.78 (0–4)	0.91 (0–4)
Walking surfaces, mean (range) (0–10)	0.83 (0–5)	0.43 (0–5)
Gait abnormality, mean (range) (0–10)	1.42 (0–7)	1.6 (0–4)
Shoes wearing, mean (range) (0–5)	0	0
- Alignment, mean (range) (0–15)	15	15

### AOFAS score

Mean AOFAS scores were plotted by treatment and time, which indicated inclining AOFAS scores in both treatment groups, see Table 2. The mean AOFAS scores in the FC at 2, 4, 6 and 8 weeks were 68.22, 82.72, 91.75 and 98.11, respectively; the corresponding values in the SLC group were 60.2, 70.20, 92.24 and 99.13. Applying the mixed-effect regression model indicated that the AOFAS score in FC were approximately 8.02 and 12.52 scores higher when compared to SLC at 2 and 4 weeks. The AOFAS score at 6 and 8 weeks showed no significant difference between the two groups. Comparing within treatment group effects indicated significantly increasing AOFAS scores after receiving treatment in both groups at each distinct time of follow up (see Table 3).

**Table 2:** Mean of VAS and AOFAS compared between two groups at 1 month to 6 months follow up

Follow up time	Treatment		Mean differences between groups	95%CI	*P*-value
	Foot cast	Short leg cast			
VAS					
2 weeks	1.97	2.91	− 0.94	− 1.53, − 0.34	0.002
4 weeks	0.58	1.23	−0.65	−1.24, − 0.05	0.033
6 weeks	0.17	0.37	−0.20	−0.78, 0.39	0.509
8 weeks	0.08	0.11	−0.03	−0.63, 0.57	0.922
AOFAS					
2 weeks	68.22	60.20	8.02	3.74, 12.1	< 0.001
4 weeks	82.72	70.20	12.52	8.27, 16.78	< 0.001
6 weeks	91.75	92.24	−0.49	−4.72, 3.74	0.820
8 weeks	98.11	99.13	−1.02	−5.30, 3.27	0.642

**Table 3:** Mean of VAS and AOFAS within group at difference time compared after baseline to 8 weeks follow up

Time	Foot cast			Short leg cast		
	Mean	Within group differences	*P*-value	Mean	Within group differences	*P*-value
VAS						
0 week	7.36	–	–	6.09	–	–
2 weeks	1.97	−5.39	< 0.001	2.91	−3.18	< 0.001
4 weeks	0.58	−1.39	< 0.001*	1.23	−1.68	< 0.001
6 weeks	0.17	−0.42	0.142	0.37	−0.86	0.003
8 weeks	0.08	−0.08	0.769	0.11	−0.25	0.382
AOFAS						
0 week	33.58	–	–	32.6	–	–
2 weeks	68.22	34.64	< 0.001	60.20	27.60	< 0.001
4 weeks	82.72	14.50	< 0.001	70.20	10.00	< 0.001
6 weeks	91.75	9.03	< 0.001	92.24	22.04	< 0.001
8 weeks	98.11	6.36	0.002	99.13	6.89	0.001

*statistically significant difference (*P* < 0.01)

### Pain VAS score

Mean VAS scores were plotted by treatment and time, which indicated declining VAS scores in both treatment groups, see Table 2. The mean VAS scores in the FC at 2, 4, 6 and 8 weeks were 1.97, 0.58, 0.17 and 0.08 respectively; the corresponding values in the SLC group were 2.91, 1.23, 0.37 and 0.11. Applying the mixed-effect regression model indicated that the VAS score in FC were approximately 0.94 and 0.65 scores lower when compared to the SLC at 2 and 4 weeks. The AOFAS score at 6 and 8 weeks had no significant differences between the two groups. Comparing within treatment group effects indicated significantly decreasing AOFAS scores after receiving treatment in both groups at each distinct time. None of the participants experienced cast related adverse effects, delayed union, nonunion and re-fracture.

## Discussion

This study is a single-blind RCT comparing FC and SLC for treatment of pseudo-Jones metatarsal fracture. This study demonstrated that the application of FC can improve pain VAS and AOFAS function at 2 and 4 weeks in the treatment of pseudo-jones metatarsal fractures when compared to SLC. However, there was no statistically significantly difference between the two groups at 6 and 8 weeks. In term of adverse effects, there were no incidents of cast related complications, delayed union, nonunion or re-fracture in both groups.

According to P. Monteban et al. [26] for the treatment strategies of pseudo-Jones metatarsal fracture can safely be treated non-operatively (full casting, backslab or bandaging) with good patient-reported outcome, less complications and re-interventions, lower healthcare cost, and without increased economic burden. Surgery can be reserved for those with delayed union after failed conservative treatment [26, 27]. There is a paucity of literature regarding non-operative treatment of Pseudo-Jones fractures of the fifth metatarsal base. Only five comparative studies [2, 14, 17–19] and one meta-analysis [20] were published and the results suggested that foot splinting (boot splint and compression bandage) resulted in higher foot functional scores when compared to short leg casting. This could be due to the foot splinting group (less rigid immobilization) taking their splint off at night, allowing ankle movement, which may have improved early functional outcomes. However, in the study [20] there has been no pain assessment, which is an important outcome in fracture treatment. There has been only one previous comparison study that assessed pain and the results suggested that pain and function recovered earlier in patients treated with a walking boot than in those with a short-leg cast [17]. However, the cost of treatment is a very important consideration because the use of the walking boot is not covered by Thai healthcare organizations (insurance, social security insurance and universal coverage) in order to reduce the cost of treatment. The walking boot cost is estimated to be 8000–10,000 baht (180–220 euros) each and one boot should cover the entire duration of treatment. On the other hand, the total cost of materials required in order to apply a single short-leg cast was estimated to be 600–1000 baht, including a Tubigrip support bandage, plaster and plaster slipper and all patients require at least 1 further cast change. Although the study [17] showed that the patients who used the walking boot recovered 3 weeks earlier and returned to work 8 days earlier when compared to cast immobilization, the treatment is much more costly, which is why the walking boot is often not used in clinical practice in some developing countries. This is the first study investigating pain and a functional outcome of FC, which ensures rigid immobilization of the pseudo-jones fracture and allows normal ankle movement when compared to SLC. This study demonstrated that pain and function recovered earlier in patients treated with a FC than in those with a SLC. Our study showed that patients achieved radiographic union by 8 weeks in both groups.

### Strengths

To the best of our knowledge, this is the first randomized controlled trial to assess the outcomes including pain (VAS), foot function (AOFAS) and complications of FC versus SLC in pseudo-Jones metatarsal fracture with 8 weeks follow-up. The follow up was reasonably high, at 100 and 97% in FC and SLC groups respectively. An intention to treat analysis was applied by considering all patients in the groups to which they were originally randomly allocated, thus minimizing bias. Radiographic evaluation at 8 weeks in all participants was done, so the incidence of fracture union was assessed to be 100%.

### Limitations

This study done to evaluate the use of foot cast and short leg cast and some participants had NSAIDs while some participants did not. Although the co-intervention effect might be influence to change the outcome VAS, AOFAS scales and bony union however additional pain medication was also similar in both groups. The sample size calculation was computed to assess primary outcomes between groups but may not be generalizable to assess secondary outcomes, therefore the statistical insignificance may be due to the risk of type II errors.

## Conclusion

This study demonstrated that the application of a foot cast can improve pain VAS and AOFAS function at 2 and 4 weeks in the treatment of pseudo-jones metatarsal fractures when compared to short leg casting. However, there was no statistically significant difference between the two groups at 6 and 8 weeks. All participants have 100% incidence of radiographic union in both groups. Cost-effective analysis should be done to compare FC and SLC in the future.

## Data Availability

Please contact author for data requests.

## Abbreviations

AOFAS: American Orthopedic Foot and Ankle Score
FC: Foot casting
IP: interphalangeal
ITT: intention-to-treat
MTP: metatarsophalangeal
NSAIDs: nonsteroidal anti-inflammatory drugs
RCT: Randomized controlled trial
SD: Standard deviation
SLC: Short leg casting
VAS: Visual analog score
